# Genomic Analysis of Adaptability and Genetic Structure of Jabal Akhdar Goats: Evidence of Positive Selection in an Indigenous Omani Breed

**DOI:** 10.3390/biology14070761

**Published:** 2025-06-25

**Authors:** Zainab Mohammad, Hussain Bahbahani, Ahmad Alfoudari, Kaadhia Al Kharousi, Al Abeer Al Hamrashdi, Al Ghalya Al Toobi, Mohammad Al Abri

**Affiliations:** 1 Department of Biological Sciences, Faculty of Science, Kuwait University, Sh. Sabah Al-Salem Campus, Al-Shadadiya, P.O. Box 5969, Safat 13060, Kuwait; zainabahmed923@gmail.com (Z.M.); ahmad.alfoudari@grad.ku.edu.kw (A.A.); 2 Department of Animal and Veterinary Sciences, Sultan Qaboos University, Muscat 123, Oman; kaadhia@squ.edu.om (K.A.K.); abeern@squ.edu.om (A.A.A.H.); alghalya@squ.edu.om (A.G.A.T.)

**Keywords:** hypoxia adaptation, inbreeding, mountain goats, runs of homozygosity, SNP genotypes

## Abstract

Jabal Akhdar goats, native to Oman’s high-altitude Jabal Akhdar mountain range, are recognized for their high growth rate, remarkable twinning rate, and adaptability to harsh environmental conditions. This study assesses the genetic structure, inbreeding levels, effective population size (*N_e_*), and linkage disequilibrium (LD) of Jabal Akhdar goats while identifying genomic regions under positive selection that may contribute to their environmental adaptation. The SNP genotypes from 72 Jabal Akhdar goats and two desert breeds from Egypt (153 Barki and 60 Saidi) revealed a clear genetic distinction between both groups. Within the Jabal Akhdar goats, genetic differentiation was also identified among the three sampled villages, indicating a village-specific genetic structure. The Jabal Akhdar breed exhibited a moderate level of inbreeding (*F*_ROH_ = 0.16), greater than that of the Barki and Saidi breeds. Additionally, Jabal Akhdar goats displayed greater LD and lower *N_e_* levels compared to the Egyptian breeds. Analysis of runs of homozygosity (ROH) and extended haplotype homozygosity-based statistics (*iHS* and *Rsb*) identified 93 genomic regions exhibiting signatures of positive selection (80 from ROH, 5 from *iHS*, and 8 from *Rsb*). These regions harbor genes associated with traits essential for environmental adaptability, including hypoxia tolerance (*SUCNR1*, *ANGPTL1, MITF, MTUS2*), muscle development and function (*MBNL1*, *ACTC1*, *CAPN5*), fertility (*GNRHR*, *CCNA1*, *SPAG1*), UV radiation resistance (*UVRAG*, *BRCA1*), bone development (*SOST*, *MEOX1*), and lipid metabolism for energy utilization (*DGAT2*, *G6PC*, *SUCLG2*). The results of this study provide valuable insights for identifying causative variants and haplotypes underlying the Jabal Akhdar goat’s superior adaptability. These findings can guide breeders in designing conservation strategies and improving the productivity of this unique indigenous breed.

## Simple Summary

Goats are a vital source of food and income for people living in harsh environments, particularly in mountainous and dry regions. In Oman, a unique breed of goat lives in the high-altitude Jabal Akhdar mountain range, where temperatures can drop below freezing and vegetation is scarce. These goats are known for their ability to survive and grow well under such difficult conditions, but little is known about the genetic traits that help them do so. In this study, we examined the genetic makeup of these goats to understand how they have adapted to their environment over time. We looked for signs of inbreeding and explored which genes may be responsible for helping the goats adapt to low oxygen levels, limited food, and temperature extremes. We also compared their genetic patterns to other desert-adapted goat breeds from Egypt. Our results identified key genetic regions that may play a role in traits like growth, reproduction, and resistance to environmental stress. This information is valuable for designing breeding programs to preserve and improve these local goat breeds. Understanding how animals adapt to tough conditions can help communities raise highly productive livestock and ensure food security in challenging climates.

## 1. Introduction

Goats constitute a vital resource for a significant proportion of the global population, particularly in low-income countries, where 90% of the global goat population resides [1]. Their resilience and versatility to produce milk, meat, and fiber make them indispensable assets for livestock owners in these regions. Furthermore, goats exhibit remarkable adaptability to a variety of environmental conditions, including the ability to forage on low-quality and woody plant species that are unsuitable for sheep and cattle [2]. A key factor in the adaptability of goats to arid environments is their array of physiological characteristics. For instance, goats can conserve water by reducing losses in urine during prolonged droughts, enabling them to efficiently withstand dehydration [2,3]. Additionally, they have relatively lower metabolic requirements and can reduce their overall metabolic activity, which enables them to survive on limited energy intake in nutrient-scarce pastures [4].

Barki and Saidi are examples of the main indigenous goat breeds in Egypt. These two genetically distinct breeds show superior resilience and adaptability to harsh, arid environmental conditions and limited vegetation [5,6]. Barki goats populate the Coastal Zone of the Western Desert in Bog Arab and Matrouh [7], while the Saidi goats are concentrated in Upper Egypt at Assuit and Aswan [7]. Indigenous goat breeds exhibit remarkable adaptation to local environmental conditions, a result of millennia of natural selection [4]. Among these, Jabal Akhdar goats (*Capra hircus*) inhabit the Jabal Akhdar mountain range in the Sultanate of Oman, surviving at altitudes between 2800 and 3000 m above sea level [8]. This breed demonstrates exceptional resilience to the region’s harsh climatic fluctuations, enduring winter temperatures that can drop below 0 °C, extremely limited rainfall averaging ~ 1 mm annually, and dry conditions prevailing on 95% of the days [8]. Studies stated that the Jabal Akhdar goats constitute a range between 5% [9] and 20% [10] of the total goat population of Oman; however, the effective population size was not determined yet and might be influenced by positive selection pressures. Notably, Jabal Akhdar goats exhibit superior growth rates compared to other breeds and rank second among Omani goat breeds in terms of twinning rates [11]. These traits, combined with their adaptability, make Jabal Akhdar goats the most valuable breed in Oman, with male bucks fetching prices as high as 2600 USD [12].

A significant concern associated with indigenous goat breeds is their level of inbreeding, defined as mating between parents that share one or more common ancestors [13]. This evolutionary mechanism is directly linked to a reduction in the genetic diversity within the breed, consequently impeding overall fitness through a mechanism known as inbreeding depression [14]. To quantify inbreeding levels, several statistical measures have been developed, among which the inbreeding coefficient (*F*_ROH_) is considered highly effective [13]. The *F*_ROH_ estimate has been extensively applied to evaluate inbreeding levels across various livestock species, including cattle [15,16], sheep [17], pigs [18], and goats [19,20,21]. This coefficient is derived from the pattern of runs of homozygosity (ROH), which are continuous segments of homozygous genotypes within the genome of individuals [22].

Natural selection is an evolutionary process that has profoundly shaped the genome of livestock species. Advances in SNP genotyping technologies and next-generation sequencing platforms have facilitated the investigation of genomic signatures of positive selection across a wide range of livestock species, including cattle [23,24,25], sheep [26,27], camels [28,29,30], and goats [31,32]. Genomic studies using SNP genotypes have defined several regions with signatures of positive selection. These include a variety of goat breeds distributed globally [31] as well as indigenous goat breeds from China [33,34] and Africa [32,35]. The genomic regions under selection harbor genes related to multiple biological functions, such as reproduction [27,31], immune response [32], weight [34], efficiency of food conversion [31], and milk production [32].

Research on the high-altitude adaptations in goats has predominantly focused on Tibetan cashmere goats in China. Using diverse genomic approaches, including exome sequence [36], whole-genome sequence [37], and SNP genotypes [38], these studies have revealed signatures of selection related to their high-altitude adaptations. Genes associated with cardiovascular system development, oxygen sensing, and hypoxia adaptation, such as endothelial PAS domain protein 1 (*EPAS1*), have been shown to be under selection in this goat breed. More recently, Zhong et al. [39] utilized SNP genotyping through the Illumina GoatSNP50 BeadChip to explore the genotypes of Tibetan goats, identifying signatures of selection in the adipogenesis regulatory factor (*ADIRF*) gene. This gene is involved in controlling fat deposition, which is crucial for survival and adaptation in high-altitude conditions.

The unique physiology and adaptability of Jabal Akhdar goats establish them as an excellent candidate to uncover the genetic mechanisms underlying high-altitude and cold-temperature adaptations. This study aims to investigate the genome of this mountain goat breed from Jabal Akhdar in Oman to assess their genetic structure and diversity, inbreeding level, extent of linkage disequilibrium, and effective population size using SNP genotype data. Furthermore, the research will explore the signatures of positive selection across their genome to define candidate regions and genes potentially associated with high-altitude adaptability. The findings from this study are expected to aid in designing informative breeding programmes aimed at conserving the diversity of this indigenous goat breed and improving its productivity.

## 2. Materials and Methods

### 2.1. Goat Populations SNP Genotype Data

A total of 3 mL of whole blood samples were collected from 72 female goats across three different villages within the Jabal Akhdar mountain range in Oman, where Jabal Akhdar goats are mainly populated: 32 goats from Hililat (23°08′03.0″ N, 57°33′35.5″ E), 19 goats from Shinoot (23°06′58.7″ N, 57°39′30.5″ E), and 25 goats from Ghaliel (23°06′17.4″ N, 57°33′48.9″ E). The approximate straight-line distances between the villages are as follows: Hililat–Ghaliel (~3.27 km), Hililat–Shinoot (~10.29 km), and Ghaliel–Shinoot (~9.80 km). This number of samples was reached due to the limited accessibility to goat samples in the mountainous range of Jabal Akhdar. The sampling procedure adhered to ethical standards, as no euthanasia was required, and the protocol was approved by the Ethics Committee for Animal Use in Research at Sultan Qaboos University (Ref:SQU/EC-AUR/2023-2024/7). Genomic DNA was extracted from the blood samples using the Puregene Blood Core Kit C (Qiagen, Germantown, MD, USA, Cat. No. 158026) and genotyped using the Illumina GoatSNP50 BeadChip v.2 at Neogen (GeneSeek, A Neogen Company, Lincoln, NE, USA). SNP genotype data for two desert goat breeds from Egypt, Barki (*n* = 153) and Saidi (*n* = 60), were obtained from the same genotyping array and retrieved from Colli, Milanesi [40].

After excluding SNPs that were unmapped or located on sex chromosomes, quality control (QC) analyses were performed on a total of 50,574 SNPs spanning 29 autosomes, with an average gap size of 48.7 kb (±33.9) (Figure S1), using Plink 1.9 [41]. The QC was conducted separately for: a combined dataset comprising Jabal Akhdar, Barki, and Saidi breeds (dataset 1) and Jabal Akhdar goats only (dataset 2). A total of 25 Barki and 1 Jabal Akhdar goat with a genotyping call rate < 95% were excluded. Furthermore, two additional Jabal Akhdar goats were excluded from downstream analyses due to a high identity-by-state (IBS > 95%) with other samples. SNPs with a genotyping call rate < 100% or significantly deviating from the Hardy-Weinberg Equilibrium (Fisher exact test *p*-value < 1 × 10^−6^) were excluded. Additionally, SNPs with a minor allele frequency ≤0.05 were further excluded for the genetic structure and signatures of selection analyses. For genetic structure analyses, SNPs in a pair of high linkage disequilibrium (LD) (r^2^ > 0.5) were also excluded (Table 1).

### 2.2. Genetic Structure Analyses

Principal Component Analyses (PCA) were conducted on the genotyped goat samples from both datasets using the *prcomp* function within the GenABEL package version 1.8-0 [42] in R software version 4.4 [43]. Likelihood-based clustering analyses were carried out on both datasets using ADMIXTURE 1.23 [44]. The number of clusters (K) ranging from 1 to the total number of populations (K = 1–5 for dataset 1 and K = 1–3 for dataset 2) was assumed, with 200 bootstrap iterations performed in each cluster. The optimal number of clusters was identified as the K value with the lowest cross-validation error. Visualizations of the PCA and admixture analyses were generated using the ggplot2 package version 3.5.2 [45] for R. Mean observed homozygosity (Ho) values for the various goat breeds were calculated using the *hom* function implemented in the GenABEL package version 1.8-0 for R software version 4.4. To assess significant differences in Ho values between goat breeds, the two-sample Mann-Whitney U test was applied.

### 2.3. ROH and Inbreeding Level Estimation

Runs of homozygosity segments in goat samples were defined using a consecutive SNP-based method implemented in the detectRUNS R package version 0.9.6 (https://cran.r-project.org/web/packages/detectRUNS/vignettes/detectRUNS.vignette.html, accessed on 22 June 2025). The analysis was performed with the following parameters: (1) a minimum of 15 SNPs per run (*minSNP = 15*), (2) a maximum allowance of two SNPs with opposing genotypes within a run (*maxOppRun = 2*), (3) a maximum of one missing SNP permitted per run (*maxMissRun = 0*), (4) a maximum distance of 1 Mb between consecutive SNPs in a run (*MaxGap = 10^6^*), and (5) a minimum run length of 1 kb (*minLengthBps = 1000*). The *F*_ROH_ value for each goat sample was calculated as the sum of the individual’s total ROH length divided by the total length of autosomes, according to McQuillan et al. [22]. This estimation was performed using the *Froh_inbreeding* function implemented in the detectRUNS package. To assess the inbreeding levels across breeds, the mean *F*_ROH_ value for each goat breed was calculated. Statistical differences in *F*_ROH_ values between goat breeds were evaluated using a two-sample Mann-Whitney U test.

### 2.4. LD and N_e_ Analyses

To measure the linkage disequilibrium (LD) level between variants, pairwise square correlation coefficient (r^2^) values were calculated for the filtered genotyped SNPs of the goat breeds, separately, using the *r*^2^ command in PLINK 1.9. Analysis parameters included a pairwise threshold of r^2^ = 0.099 (*ld-window-r2 0.099*) and a maximum LD window size of 1000 kb (*ld-window-kb 1000*). The extent of LD decay was assessed for the three goat breeds of dataset 1 by analyzing bins spanning set inter-variant distances. The *N_e_* for the Jabal Akhdar, Barki, and Saidi goat breeds was estimated using SNeP version 1.1 [46] based on the pairwise LD estimates. Default parameters were employed, except for the minimum r^2^ threshold for SNP pairs, which was set at 0.099 (*minr2 = 0.099*).

### 2.5. Signatures of Selection Analyses

Two intra-population analyses, namely ROH and the integrated Haplotype Score (*iHS*) [47], along with a single inter-population analysis (*Rsb*) [48], were conducted on the mountain and desert goat breeds. The *Rsb* statistic detects recent selection events by comparing the extended haplotype homozygosity (EHH) between populations, making it complementary to *iHS*, which identifies incomplete selective sweeps within a single population [47,48]. Candidate regions exhibiting signatures of positive selection in the Jabal Akhdar mountain goat breed were identified and compared with regions identified in desert breeds. ROH regions observed in 50% or more of the goat samples were classified as candidate regions, as followed by Santos et al. [49], and peaks were subsequently merged to define the coordinates of the ROH islands using the *merge* function of BEDTools version 2.28 [50]. Individual haplotypes were phased using Beagle 5.4 [51] using default parameters. The extended haplotype homozygosity (EHH)-based statistics, *iHS* and *Rsb*, were calculated using the rehh package version 3.2.2 [52] in R software version 4.4. The *Rsb* values were calculated by comparing the natural logarithm of the ratio of integrated EHHS (site-specific EHH) for each SNP (iES) between the Jabal Akhdar goats (mountain breed) and the combined Barki and Saidi goats (desert breeds). The *iHS* analysis was performed exclusively on the Jabal Akhdar goats after excluding SNPs with a minor allele frequency of less than 0.05. For each genotyped SNP, the natural logarithm of the ratio between the integrated EHH for the reference allele (iHHR) and the alternative allele (iHHA) was computed. To avoid SNP-specific variations, the raw *Rsb* and *iHS* values were subsequently averaged in sliding windows of 15 SNPs (average window size = 1 Mb), with a step size of 7 SNPs. These window-based values were then converted to *p*-values based on fractional ranks using the *stat_to_pvalue* function in the MINOTAUR package version 0.0.1 [53] in R software version 4.4. The *p*-values were transformed into rank-based *p*-values using either a right-tailed test (for *Rsb*) or a two-tailed test (for *iHS*). In both analyses, a threshold of −log_10_ (*p*-value) equal to 2, corresponding to the extreme 1% tail of the empirical distributions for both statistics, was used to define significant windows. Candidate *iHS* and *Rsb* regions were established if at least two consecutive windows, separated by no more than 100 kb, the distance at which the r^2^ value in Jabal Akhdar goats declined below 0.25, passed this threshold. The peak window in each case was used to define the candidate region coordinates.

### 2.6. Functional Characterization of the Candidate Selection Regions

Genes located within the ROH islands, *iHS*, and *Rsb* candidate regions of mountain goats were retrieved using the intersect function in BEDTools version 2.28, referencing the goat genome assembly (ARS1.2) gene list. Functional analysis of these genes was carried out with the g:GOSt tool from g:Profiler version e112_eg59_p19_25aa4782 [54], which provided insights into enriched gene ontology terms associated with biological processes and molecular functions. To account for multiple testing, the g:SCS algorithm in g:Profiler was employed to adjust *p*-values during the gene ontology and pathway enrichment analyses. The functional annotation tool implemented in Database for Annotation, Visualization, and Integrated Discovery (DAVID) Bioinformatics resource version 6.7 [55], was also used to define functionally enriched biological pathways. An enrichment score of 1.3, which is equivalent to a Fisher exact test *p*-value of 0.05, was used as a threshold to define the significantly enriched functional terms, comparing the results to the goat genome background. Finally, the biological roles of the identified genes were further investigated through a comprehensive review of relevant scientific literature.

## 3. Results

### 3.1. Genetic Structure Analyses

**Figure 1:**
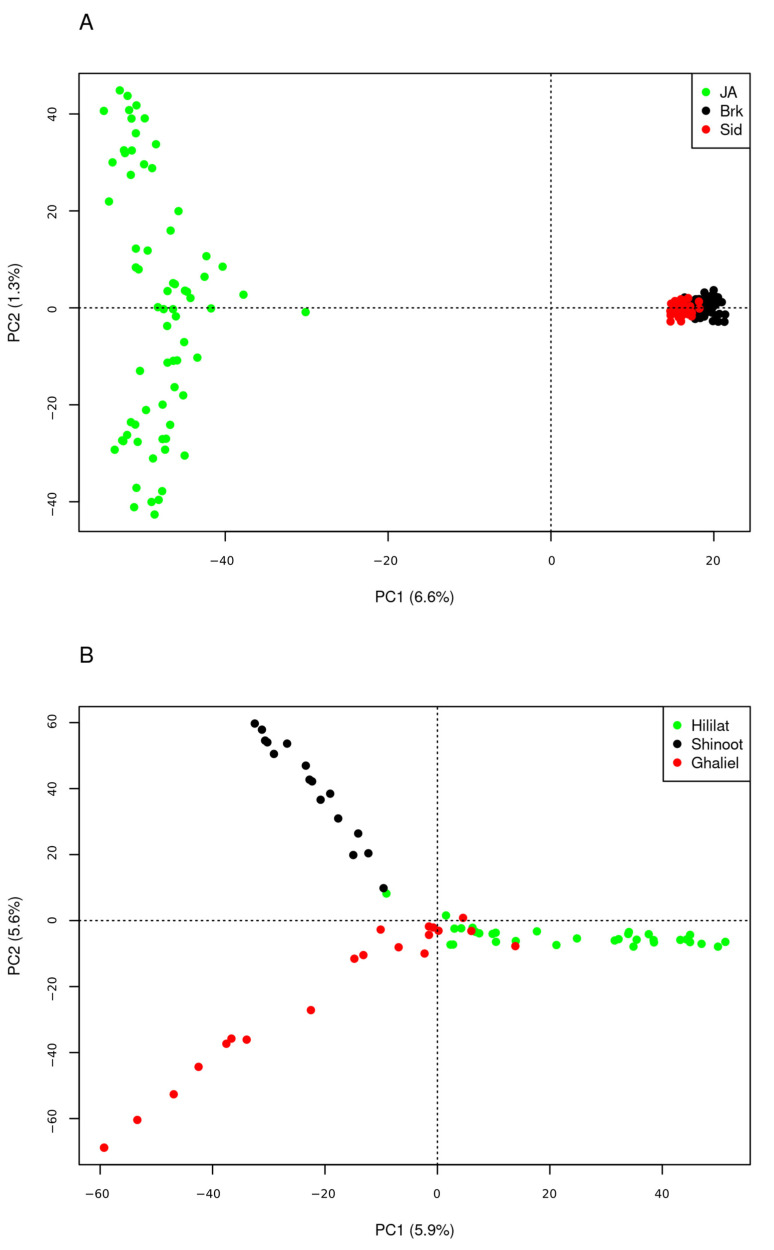
PCA plots of the first and second principal components (PC) for (**A**) Jabal Akhdar (JA), Barki (Brk), and Saidi (Sid) goat breeds (dataset 1), and (**B**) Jabal Akhdar goat populations (Hililat, Shinoot, and Ghaliel) (dataset 2).

**Figure 2:**
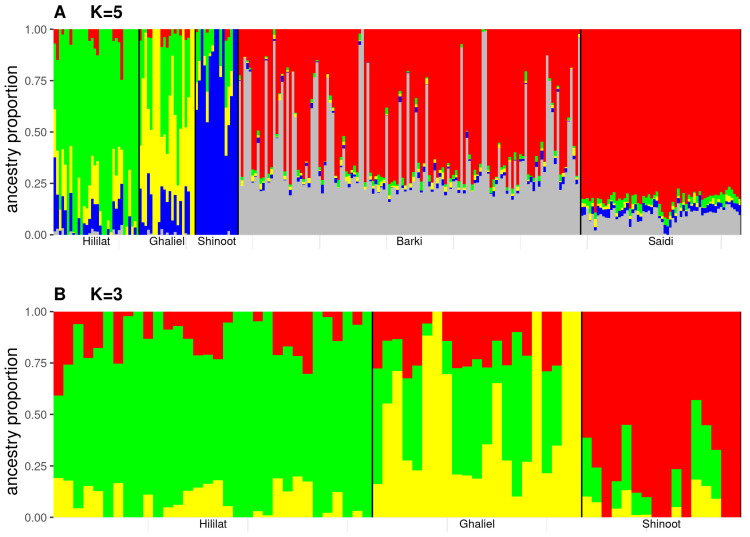
Admixture analysis of goat breeds based on SNP data. (**A**) Admixture plot for K = 5 on Jabal Akhdar (Hililat, Shinoot, and Ghaliel), Barki, and Saidi goat breeds (dataset 1), and (**B**) admixture plot for K = 3 Jabal Akhdar goat populations (Hililat, Shinoot, and Ghaliel) (dataset 2). Each Individual is represented by a thin vertical line, partitioned into colored segments that correspond to their estimated membership fractions in K = 5 (**A**) or K = 3 (**B**). The X-axis lists the studied goat breeds. The Y-axis represents the estimated ancestry proportion (ranging from 0 to 1).

The PCA of dataset 1 revealed a genetic distinction between Jabal Akhdar goats and the Barki and Saidi breeds along the first principal component, which accounted for 6.6% of the total genetic variation. A degree of genetic heterogeneity was observed among the Jabal Akhdar goats via the second principal component (Figure 1A). The third principal component, which accounted for 1.2% of the total variation, showed a degree of genetic distinction between Barki and Saidi goats (Figure S2). When dataset 2 was analyzed, which focused on the Jabal Akhdar goat breed alone, a distinct genetic separation between village groups was observed across the first two principal components (Figure 1B). Admixture analysis on the different goat breeds in dataset 1 indicated K = 5 is the optimal number of clusters (Table S1). At this K value, the desert goat breeds (Barki and Saidi) were differentiated from the mountain goats, and a degree of genetic distinction was observed between Barki and Saidi goats, highlighting a substantial level of introgression. Furthermore, genetic separation was also revealed among the three goat populations from Jabal Akhdar, with a high degree of genetic admixture between them, particularly between Hililat and Ghaliel goats (Figure 2A). Similar genetic clustering has also been identified in Jabal Akhdar goats separately (dataset 2) at a K value of 3 (Figure 2B), which is the optimal number of clusters between them (Table S1). The mean ± standard deviations (SD) Ho value of the Jabal Akhdar goat breed was 0.65 (± 0.018), which was significantly greater than that of the Barki 0.59 (± 0.02) and Saidi 0.61 (± 0.04) breeds (*p*-value < 0.01). No significant variation was observed among the mean Ho values of Jabal Akhdar goats from the three different villages (*p*-value > 0.01): 0.61 (± 0.02) for Hililat, 0.62 (± 0.02) for Shinoot, and 0.61 (± 0.001) for Ghaliel.

### 3.2. Detection of ROH Segments and Estimation of F_ROH_

**Figure 3:**
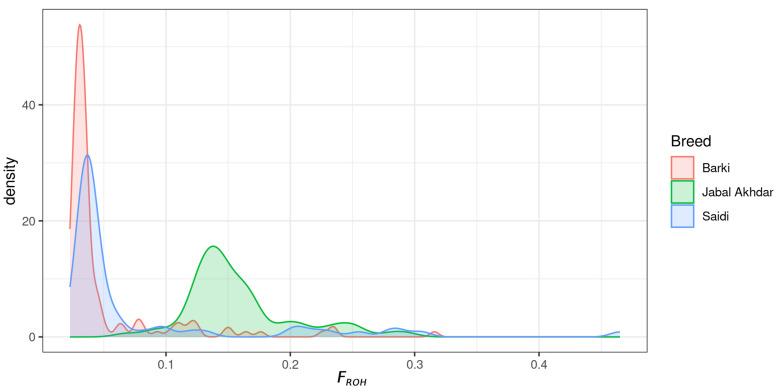
Density plot illustrating the distribution of the inbreeding coefficient (*F*_ROH_) values across the genome of the different goat breeds included in the study.

A total of 11,342 ROH segments were identified in Jabal Akhdar goats, which were classified into five distinct size categories: 0–2 Mb (69%), 2–4 Mb (21%), 4–8 Mb (7%), 8–16 Mb (2%), and >16 Mb (2%) (Figure S3). A significantly higher mean ± SD genome-wide *F*_ROH_ estimate (*p*-value < 0.01) was observed for the Jabal Akhdar goat breed compared to the Barki and Saidi breeds: 0.16 (±0.04) for Jabal Akhdar, 0.05 (±0.05) for Barki, and 0.08 (±0.09) for Saidi goats (Figure 3; Figure S4 and Table S2). Notably, higher mean chromosome-wise *F*_ROH_ estimates were obtained for Jabal Akhdar goats than the other two desert goat breeds over all autosomes (Figure S4). Within the Jabal Akhdar group, the mean ± SD genome-wide *F*_ROH_ values were comparable among the three goat populations with no significant differences between them (*p*-value > 0.01): 0.16 (±0.05) for Hililat, 0.18 (±0.05) for Ghaliel, and 0.17 (±0.02) for Shinoot goats.

### 3.3. LD and N_e_ Analyses

**Figure 4:**
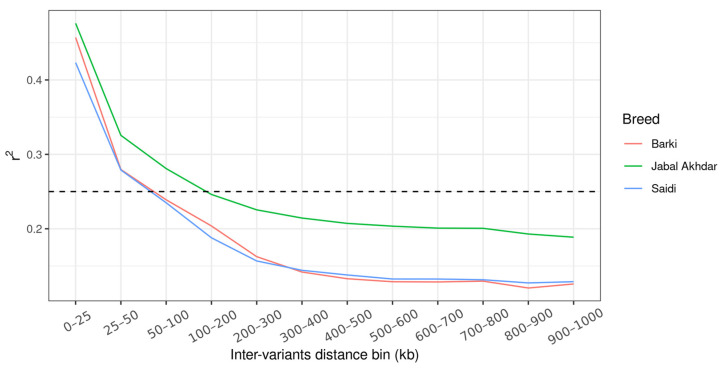
The extent of LD decay measured by the square correlation coefficient (r^2^) across the autosomes of Jabal Akhdar, Barki, and Saidi goats. The dashed line threshold is at r^2^ = 0.25.

**Figure 5:**
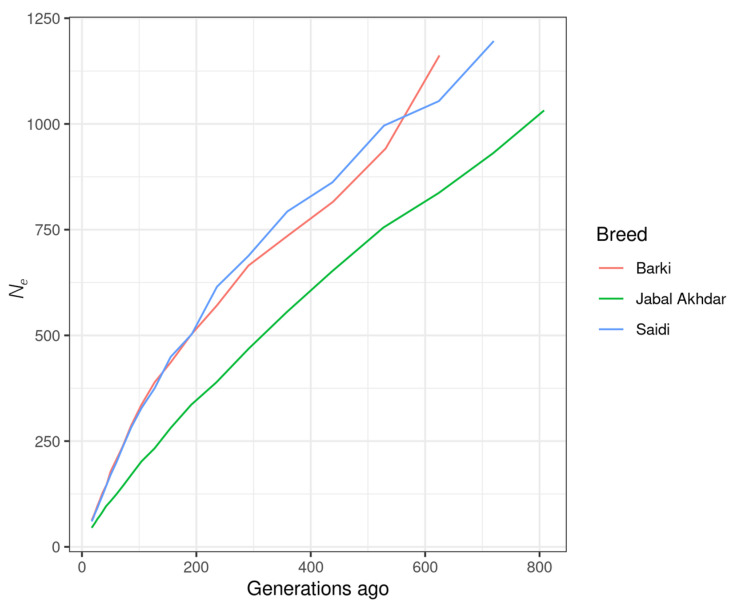
Effective population size (*N*_e_) estimations for Jabal Akhdar, Barki, and Saidi goat breeds.

As an estimation of the goats LD level, the mean ± SD r^2^ values for the Jabal Akhdar, Barki, and Saidi goats were 0.23 (±0.15), 0.22 (±0.18), and 0.2 (±0.15), respectively. The greatest r^2^ values across the three breeds were observed on chromosome 12, while the lowest values were on chromosomes 19 and 9 for the Jabal Akhdar goats and the other two desert breeds (Barki and Saidi), respectively (Figure S5). The extent of LD in Jabal Akhdar goats was found to be greater than that of the other two goat breeds, with r^2^ values declining below 0.25 at inter-variant distances ≥100 kb in Jabal Akhdar goats and ≥50 kb for the Barki and Saidi breeds (Figure 4). Effective population size estimates for all three goat breeds showed a declining trend over the past 800 generations, with Jabal Akhdar goats exhibiting a lower *N_e_* compared to the Barki and Saidi breeds (Figure 5).

### 3.4. Candidate Selection Regions and Functional Characterization

**Figure 6:**
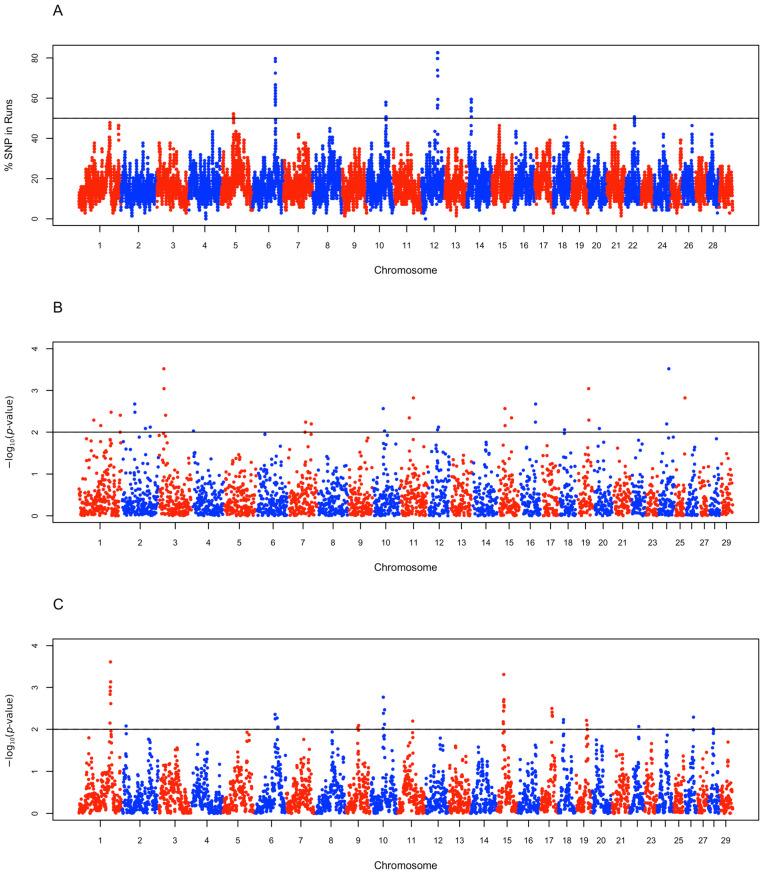
Manhattan plots of genome-wide selection scans. (**A**) ROH islands in Jabal Akhdar goats with each data point representing a genomic window showing high prevalence (≥50%) of overlapping ROH segments across individuals. (**B**) *iHS* analysis on the Jabal Akhdar goats with each point representing a genomic window (15 SNPs). The y-axis showing −log_10_ rank-based *p*-values of two-tailed test where a value of 2 was set as a significance threshold to define candidate SNPs/regions under selection (5 candidate regions). (**C**) *Rsb* analysis between Jabal Akhdar goats and the two desert goat breeds (Barki and Saidi) with each point representing a genomic window (15 SNPs). The y-axis showing −log_10_ rank-based *p*-values of right-tailed test where a value of 2 was set as a significance threshold to define candidate SNPs/regions under selection (8 candidate regions).

In examining ROH regions shared by ≥50% of Jabal Akhdar goat samples, 80 regions were identified. These regions were subsequently merged into six ROH islands on chromosomes 5, 6, 10, 12, 14, and 22 (Figure 6A and Table S3). Notably, the islands on chromosomes 6 and 12 were also observed in the desert goat breeds (Figure S6A and Table S4). A total of five candidate genomic regions potentially under positive selection were identified in Jabal Akhdar goats using the *iHS* statistic, located on chromosomes 2, 3, 15, 16, and 19 (Figure 6B and Table S3). None of these regions overlapped with the desert goats’ candidate *iHS* regions (Figure S6B and Table S4). The *Rsb* analysis comparing Jabal Akhdar to the desert goat breeds identified eight genomic regions under selection: one region in chromosomes, located on chromosomes 1 (one region), 6 (two regions separated by 9.5 Mb), 10 (two regions separated by 3.8 Mb), 15 (one region), 17 (one region), and 18 (one region) (Figure 6C and Table S3).

As summarized in Table S5, a total of 550, 116, and 62 genes were identified in the Jabal Akhdar goat breed within the genomic regions flagged by ROH, *iHS*, and *Rsb* analyses, respectively. These regions represent candidate loci under positive selection and may harbor genes associated with traits relevant to environmental adaptability. Functional profiling of these genes, conducted using g:GOSt in g:Profiler, revealed significant enrichment in several molecular and biological processes. Key pathways included lysozyme activity, salivary secretion, peptidoglycan muralytic activity, tyrosine activity, and developmental processes (Table S6). A total of 45 functionally enriched clusters were defined by the DAVID analysis, with four of them being significantly enriched. These clusters were associated with lysozyme activity (enrichment score = 5.68), tyrosine metabolism (enrichment score = 4.48), milk proteins (enrichment score = 3.22), peptidase (enrichment score = 3.17), and glutathione hydrolase activity (enrichment score = 1.86) (Table S7). A literature review further highlighted the biological significance of the retrieved candidate genes, linking them to key biological functions, including hypoxia tolerance, muscle development and function, fertility, UV radiation resistance, bone development, and lipid metabolism for energy utilization (Table 2).

## 4. Discussion

Analyzing the genomes of Jabal Akhdar mountain goats from Oman and two desert goat breeds from Egypt, Barki and Saidi, revealed a geographical-wise genetic distinction between them. Within the Jabal Akhdar population, a degree of genetic separation has also been observed corresponding to their sampling villages, with a substantial level of genetic admixture. Higher genetic admixture was observed between Hililat and Ghaliel goats, possibly due to their geographical proximity. This indigenous Omani breed exhibited higher levels of inbreeding, a greater extent of LD, and a lower *N_e_* compared to the desert goat breeds. Additionally, candidate selection regions were also identified in the genome of the Omani mountain goats, harboring genes with biological functions potentially related to their natural adaptation to the surrounding environmental conditions. These genes are associated with traits such as hypoxia tolerance, muscle development and function, fertility, UV radiation resistance, bone development, and lipid metabolism for energy utilization.

A moderate inbreeding level was estimated in Jabal Akhdar goats, higher than the levels in Barki and Saidi desert breeds. This likely stems from a long-term population isolation in such a mountainous geographical range and limited gene flow with other goat breeds, rather than recent bottlenecks, which is reflected in the abundance of short ROH segments (69%) in their genomes [56]. These findings align with global observations in isolated goat populations, such as the insular Bilberry and Palmeran breeds (*F*_ROH_ = 0.22 and 0.23, respectively) [57], as well as goats from Madagascar like the Sofia (*F*_ROH_ = 0.18) and Diana (*F*_ROH_ = 0.152) breeds [20]. The elevated LD in Jabal Akhdar goats is thus plausibly driven by this historical inbreeding.

Several genomic candidate regions with signatures of positive selection were identified in Jabal Akhdar goats, potentially reflecting their adaptability to the high-altitude environments. Notably, two ROH islands (located on chromosomes 12 and 6) were also found under selection in the desert goat breeds, suggesting a shared role in the general adaptive physiologies of goats. The absence of overlap between the candidate regions identified by the different statistics employed in this study is not unexpected for different reasons. First, the haplotype-based statistics, *iHS* and *Rsb*, are better suited to detect selection events occurring over distinct time frames, primarily targeting more recent selection pressures compared to the homozygosity-based statistic ROH [58]. Second, some of the outlier SNPs identified by the ROH statistics may be a result of the higher inbreeding level in Jabal Akhdar goats, as indicated by their *F*_ROH_ value and low effective population size. Although we attempted to mitigate this issue by selecting the highly prevalent SNPs located within ROH regions, this limitation of the ROH analysis persists. However, the two haplotype-based statistics employed in our study help to overcome this challenge. Third, *iHS* possesses limited power to detect alleles or haplotypes that have reached fixation, unlike the *Rsb* statistic. Moreover, *Rsb* may fail to identify a candidate region in mountain goats if the favored haplotype has also been subjected to selection in the desert breeds [58].

The signature of selection analyses revealed numerous candidate genes that are hypothesized to be associated with functional categories related to environmental adaptation in Jabal Akhdar goats. Further validation is required to confirm the function of these genes through the inclusion of phenotypic data and experimental analyses. One example of the listed functional categories pertains to adaptations against hypoxia, which are essential for survival in high-altitude environments. Hypoxia tolerance mechanisms have been documented extensively in high-altitude populations, notably in Tibetan humans and indigenous plateau humans [59,60]. Several genes were identified as collectively involved in oxygen sensing, vascular response, and metabolic regulation under hypoxic conditions: succinate receptor 1 (*SUCNR1*) on chromosome 1, angiopoietin-like 1 (*ANGPTL1*) on chromosome 16, microphthalmia-associated transcription factor (*MITF*) on chromosome 22, and microtubule-associated scaffold protein 2 (*MTUS2*) on chromosome 12. For instance, *SUCNR1* mediates succinate signaling in the brain cortex [61,62], *ANGPTL1* is involved in hypoxia-induced neovascularization [63], *MITF* is transiently upregulated under hypoxia and regulates succinate metabolism [64], and *MTUS2* plays a role in cardiovascular development, potentially contributing to hypoxic adaptation [65].

Muscle development and function are essential for mobility, foraging, and predator evasion in rugged mountainous environments. Muscle development and function are also reported in high-altitude species, such as the Changthangi sheep from Ladakh [66] and desert-adapted dromedary camels [67]. The muscleblind-like protein 1 (*MBNL1*) gene, located on chromosome 1, is a regulator of mRNA splicing that influences muscle function. Knockout studies in mice revealed that *MBNL1* depletion results in myotonia [68]. This gene has also been identified to be under selection in Jintang Black goats [37]. Actin alpha cardiac muscle 1 (*ACTC1*), located on chromosome 10, is another key gene involved in muscle development. *ACTC1* is expressed in skeletal muscle tissues of cattle and pigs [37,69]. In humans, *ACTC1* maintains Pax7+ myogenic progenitor cells and supports muscle regeneration following injury [70]. Additionally, the calpain-5 (*CAPN5*) gene, located on chromosome 15, is essential for myoblast differentiation, as demonstrated by a mouse knockdown study [71]. In other species, *MBNL1* was found to be under selection in three different Tibetan fish groups living at high altitiudes, in comparison to low-altitude fish species, and was linked to hypoxia and energy metabolism adaptations [72]. Additionally, a study by Ji, Jiao [73] reported that the expression of *ACTC1* was upregulated in the muscle tissue of Altay sheep breeds living in high-altitude, cold environments compared to the cold-intolerant Hu sheep breeds, suggesting a possible role in thermogenesis within muscle tissue.

Structural adaptations in bone and cartilage provide essential support for mobility and endurance in steep, uneven terrains. Genes associated with bone development are also under positive selection in desert-adapted rodents and dromedary camels [30,74,75]. Sclerostin (*SOST*), located on chromosome 19, is an osteocyte-derived negative regulator of bone formation. Knockout studies in mice have revealed that loss of *SOST* results in a high-bone-mass phenotype [76]. Mesenchyme homeobox 1 (*MEOX1*), also located on chromosome 19, is essential for vertebral development. Mutations in *MEOX1* result in skeletal defects such as vertebral fusion, congenital scoliosis, and asymmetry of the pectoral girdle, as shown in a study on zebrafish [76,77].

Efficient lipid metabolism and energy utilization are vital for surviving in harsh, high-altitude, and extreme conditions. Lipid metabolism adaptations, crucial for energy storage and management, are prevalent both in high-altitude species, including Tibetan pigs and plateau lizards [78,79], and prominently in desert-adapted dromedary camels [30,80]. Diacylglycerol O-acyltransferase 2 (*DGAT2*), located on chromosome 15, is involved in triacylglycerol (TAG) synthesis through its interaction with lipid droplets. Overexpression of *DGAT2* has been shown to increase TAG content [81,82]. Glucose-6-phosphatase (*G6PC*), located on chromosome 19, encodes a key enzyme in gluconeogenesis and glycogenolysis. Deficiencies in *G6PC* lead to glycogen storage diseases in both humans and mice [83]. Additionally, the succinate-CoA ligase GDP-forming subunit beta (*SUCLG2*) gene, located on chromosome 22, has been associated with redox homeostasis and energy metabolism in pigs’ longissimus dorsi muscle [84]. *DGAT2* has also been implicated in high-altitude adaptation in other species. In yaks inhabiting elevations between 2000 and 5000 m, it exhibits high expression levels, with the greatest expression observed in subcutaneous fat tissue [85]. Additionally, another study reported that *SUCLG2* expression was downregulated in individuals susceptible to acute mountain sickness (AMS) compared to AMS-resistant individuals following exposure to hypoxia and high-altitude stress [86]. These findings suggest a possible role for *SUCLG2* in metabolic adaptation to high-altitude environments.

Genetic adaptations enhancing UV resistance are critical for mitigating the effects of intense solar radiation and reducing the risk of damage in high-altitude environments, where UV radiation is higher [87]. UV radiation resistance, critical in harsh desert environments, is also evidenced in desert-adapted mammalian species such as the Rüppell’s fox [88]. The UV radiation resistance-associated gene (*UVRAG*), located on chromosome 15, plays a role in repairing UV-induced DNA damage. In Drosophila models, deletion of *UVRAG* resulted in UV-damage hypersensitivity [68]. Additionally, BRCA1 DNA repair associated (*BRCA1*), located on chromosome 19, has been implicated in the repair of UV-induced DNA damage. Deficiency in *BRCA1* in human breast cancer cell lines increases sensitivity to UV-C irradiation [89].

Fertility is critical for sustaining population dynamics in extreme conditions. Gonadotrophin-releasing hormone receptor (*GNRHR*), located on chromosome 6, encodes a G-protein-coupled receptor essential for gametogenesis and steroidogenesis through luteinizing hormone and follicle-stimulating hormone secretion [90]. Cyclin A1 (*CCNA1*), located on chromosome 12, regulates meiosis and cell cycle progression. Disruption of *CCNA1* in male mice results in sub-fertility or sterility due to reduced sperm production [91]. The sperm-associated antigen 1 (*SPAG1*) gene, located on chromosome 14, influences oocyte meiotic execution via AMPK and MAPK signaling pathways, as shown in a study on mice [92]. Notably, *CCNA1* has also been found to be downregulated in yak–cattle F1 hybrid males compared to the yak parent, contributing to their sterility, despite the hybrids exhibiting superior adaptability to high-altitude, low-temperature, and hypoxic conditions [93].

Notably, several of the identified genes overlap with those reported in prior studies on goats, including populations adapted to high-altitude environments [36,37,38,39]. *MITF* and *MTUS2* have also been identified to be under selection in Tibetan goats inhabiting high-altitude regions [36,38]. Another study on individuals with established high-altitude pulmonary edema (HAPE) reported that *ANGPTL1* is down-regulated in response to hypoxia at high altitudes compared to individuals who are acclimatized to such conditions [94]. *MITF* has also been identified to be under selection in Tibetan pigs [95] and Ethiopian sheep [96], both inhabiting high-altitude environments. In these species, *MITF* is believed to contribute to UV radiation resistance. Specifically, it regulates melanin synthesis [95], melanocyte differentiation [97], and pigmentation [98].

This study represents the first investigation into the genetic structure, inbreeding levels, and signatures of selection in the genome of Jabal Akhdar mountain goats. However, several considerations must be addressed to enhance the validity and robustness of the findings. Although sampling Jabal Akhdar goats is logistically challenging, increasing the sample size remains crucial for more accurately defining haplotypes and estimating their frequencies in *iHS* and *Rsb* analyses. A larger sample size would enhance the statistical power of these tests, thereby reducing the likelihood of false positives and false negatives. The current sampling, limited to three villages, may also not capture the full geographic or ecological range of the breed, potentially overlooking rare alleles or localized adaptation. Expanding sampling coverage across the broader Jabal Akhdar region would provide a more comprehensive representation of the population’s genetic diversity. Additionally, as in Al-Abri and Al Kharousi [9], incorporating phenotypic data related to environmental adaptabilities for the genotyped goats is essential for identifying potential genetic associations through genome-wide association studies. Identifying potential genetic associations will pave the way for subsequent experimental validation studies aimed at determining the functional implications of the identified candidate variants. The principal findings of this study may assist goat breeders in establishing genetically informed breeding programs, thereby preserving genetic diversity in this local goat breed and conserving regions under positive selection. Additionally, these results may inform conservation strategies for other indigenous breeds inhabiting similar environments, utilizing comparative genomic studies designed to identify overlapping selection signatures.

The Illumina GoatSNP50 BeadChip used in this study is associated with two main limitations: low genome representation and an ascertainment bias towards specific goat breeds [99]. The low resolution of this genotyping array is also associated with a mean inter-variant gap size of 48.7 kb, and the minority of SNPs (< 5%) contain a gap size of more than 100 kb. As a result, certain genomic regions may be underrepresented, limiting the ability to effectively detect selection signatures in these areas. Additionally, Kardos and Åkesson [100] emphasized the need for caution when interpreting ROH results derived from datasets containing thousands of loci, as such data may lead to inaccurately defined ROH segments. Furthermore, the genotyping array’s ascertainment bias, stemming from its design based on specific goat breeds, may compromise the accuracy of assessments related to genetic diversity and relationships of indigenous goat breeds that were not included in its development. This limitation arises from the absence of breed-specific polymorphic SNPs that could effectively distinguish these populations genetically. Employing whole-genome sequence data with appropriate depth and breadth of coverage in future research could overcome these limitations and facilitate the identification of candidate regions in the Jabal Akhdar goat genome that remain undetectable with the current genotyping approach.

## 5. Conclusions

In this study, we conducted the first genomic investigation of the indigenous goat breed from Oman’s high-altitude Jabal Akhdar mountain range, focusing on signatures of selection and inbreeding levels. A moderate level of inbreeding was observed, likely a consequence of the breed’s geographic isolation. We also identified potential signatures of selection in the genome, which may be related to the breed’s adaptability to the challenging high-altitude environmental conditions. To validate these findings, further research including phenotypic data linked with their adaptability and productivity, and whole-genome sequencing is needed. The whole-genome sequence data would overcome the limitations of the current genotyping array in assessing genetic diversity and investigating signatures of selection. Incorporating phenotypic data can help in conducting genome-wide association analysis to determine potential associations between causative variants and haplotypes with the desired traits. Additionally, broader geographic sampling across the full range of the breed’s habitat is needed to fully capture its genetic diversity and detect localized adaptations. This would provide valuable insights that could support the development of genetically informed breeding programs to conserve this unique breed and improve its productivity. This study supports several United Nations Sustainable Development Goals (SDGs), including “Zero Hunger”, by laying the groundwork for improving the productivity and resilience of livestock breeds. It also contributes to “Climate Action” and “Life on Land”, by providing a basis for developing climate-adapted breeding strategies and informing efforts to conserve genetic diversity, respectively. Finally, it supports “Gender Equity”, recognizing the critical role women play in goat rearing in the Jabal Akhdar region.

## Figures and Tables

**Table 1:** Number of SNPs excluded based on various quality control criteria for the combined genotypes of Jabal Akhdar, Barki, and Saidi breeds (dataset 1) and Jabal Akhdar goats only (dataset 2).

Quality Control Criteria	Dataset 1	Dataset 2
Genotyping call rate	19,059	2126
Hardy-Weinberg Equilibrium	121	29
Minor allele frequency *	998	5589
Linkage disequilibrium **	584	7781

* Exclusion criteria for the genetic diversity and signatures of selection analyses. ** Exclusion criteria for the signatures of selection analyses only.

**Table 2:** The functional categories of candidate genes, their corresponding IDs and names, the coordinates of the respective candidate region under selection, and the identifying statistics. Please refer to Table S5 for the list of genes in the candidate regions under selection defined by this study.

Functional Category	Gene ID	Gene Name	Candidate Region Chr: Start-Stop (bp)	Identifying Statistic(s)
Hypoxia Tolerance	*SUCNR1*	Succinate receptor 1	1: 115,626,920–115,636,639	Rsb
	*ANGPTL1*	Angiopoietin-like 1	16: 58,825,678–58,849,019	iHS
	*MITF*	Microphthalmia-Associated Transcription Factor	22: 31,524,691–31,755,591	ROH
	*MTUS2*	Microtubule-Associated Scaffold Protein 2	12: 55,325,810–55,710,530	ROH
Muscle Development and Function	*MBNL1*	Muscleblind-Like Protein 1	1: 114,974,326–115,192,808	Rsb
	*ACTC1*	Actin Alpha Cardiac Muscle 1	10: 72,761,190–72,766,594	ROH
	*CAPN5*	Calpain-5	15: 26,421,548–26,478,072	iHS
Lipid Metabolism	*DGAT2*	Diacylglycerol O-Acyltransferase 2	15: 27,720,879–27,753,736	Rsb
	*G6PC*	Glucose-6-Phosphatase	19: 42,622,248–42,632,787	iHS
	*SUCLG2*	Succinate-CoA Ligase GDP-Forming Subunit Beta	22: 33,808,508–34,098,836	iHS and ROH
UV Radiation Resistance	*UVRAG*	UV Radiation Resistance-Associated Gene	15: 27,379,066–27,699,522	Rsb
	*BRCA1*	BRCA1 DNA Repair Associated	19: 42,742,613–42,809,204	iHS
Fertility	*GNRHR*	Gonadotrophin-Releasing Hormone Receptor	6: 84,144,226–84,161,854	Rsb
	*CCNA1*	Cyclin A1	12: 61,577,282–61,587,501	ROH
	*SPAG1*	Sperm-Associated Antigen 1	14: 17,990,033–18,077,090	ROH
Bone Development	*SOST*	Sclerostin	19: 43,286,595–43,291,485	iHS
	*MEOX1*	Mesenchyme Homeobox 1	19: 43,186,727–43,205,838	iHS

## Data Availability

The raw data supporting the conclusions of this article will be made available by the authors on request.

## Supplementary Materials

The following supporting information can be downloaded at: https://www.mdpi.com/article/10.3390/biology14070761/s1, Figure S1: A histogram plot of the gap size in kb between the SNPs included in the Illumina GoatSNP50 BeadChip v.2. Figure S2: PCA plot of the first and third principal components (PC) on Jabal Akhdar (JA), Barki, and Saidi goat breeds. Figure S3: Distribution of the sizes of ROH segments in each breed. Figure S4: The chromosome-wise inbreeding coefficient (F_ROH_) for the Jabal Akhdar, Barki and Saidi goat breeds. Data represent mean ± SEM. Figure S5: The chromosome-wise square correlation coefficient (r^2^) for the Jabal Akhdar, Barki and Saidi goat breeds. Data represent mean ± SEM. Figure S6: Manhattan plots of the genome-wide (A) ROH islands and (B) *iHS* analysis on combined desert goat breeds (Barki and Saidi). The significance threshold is set on ROH prevalence at 50% of the individuals. For the *iHS* analysis, the significance thresholds are set as –log_10_ (*p*-value) of 2. Table S1: The cross-validation (CV) error of the different cluster values (K) on datasets 1 and 2. Table S2: The inbreeding coefficient (F_ROH_) for the different goat breeds included in the study. JA = Jabal Al-Akhdar breed from Oman. BRK = Barki breed from Egypt. Table S3: Runs of homozygosity (ROH) islands, and candidate iHS and Rsb regions on the genome of Jabal Akhdar goats. Table S4: Runs of homozygosity (ROH) islands and iHS candidate regions on the genome of desert goat breeds. Table S5: Genes found on the ROH islands and candidate iHS and Rsb regions on the genome of Jabal Akhdar goat breed. Table S6: Functional profiling of the candidate genes on Jabal Akhdar goats based on g:Profiler software. Table S7: Functional enrichment DAVID analysis on Jabal Akhdar goats’ candidate genes.
