# Supplementary material for: Genomic Analysis of Adaptability and Genetic Structure of Jabal Akhdar Goats: Evidence of Positive Selection in an Indigenous Omani Breed
Source: Biology (Basel). 2025 Jun 25;14(7):761. doi: 10.3390/biology14070761 (PMC12292401; doi:10.3390/biology14070761)
Supplement: Supplementary file 1 [file biology-14-00761-s001.zip › Supplementary_material.pdf]

Supplementary Material

Genomic analysis of adaptability and genetic structure of Jabal Akhdar goats: evidence of positive selection in an indigenous Omani breed

Zainab Mohammad[1], Hussain Bahbahani[1*], Ahmad Alfoudari[1], Kaadhia Al Kharousi[2], Al Abeer Al Hamrashdi[2], Al Ghalya Al Toobi[2], Mohammad Al Abri[2]

[1] Department of Biological Sciences, Faculty of Science, Kuwait University, Sh. Sabah Al-Salem campus, Al-Shadadiya, Kuwait

[2] Department of Animal and Veterinary Sciences, Sultan Qaboos University, Muscat, Oman

Correspondence:

Hussain Bahbahani

hussain.bahbahani@ku.edu.kw)

Mohammad Al Abri

abri1st@squ.edu.om

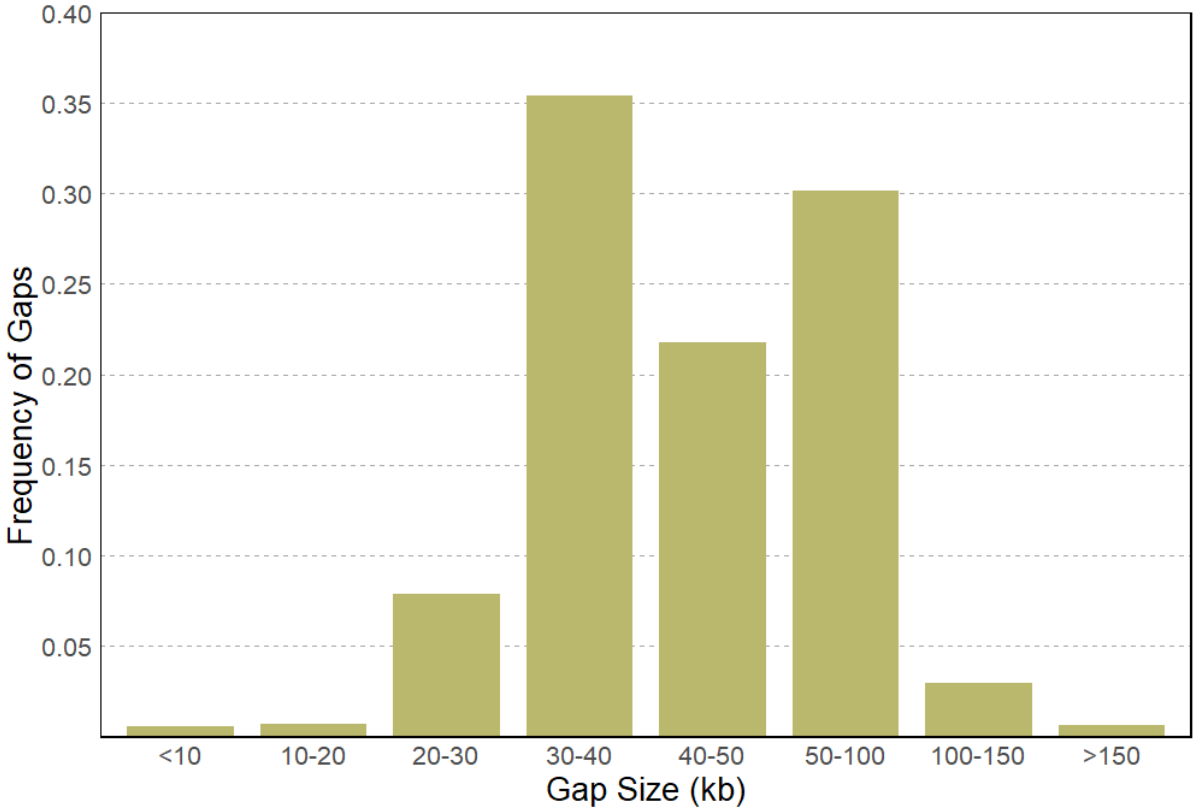

Figure S1: A histogram plot of the gap size in kb between the SNPs included in the Illumina GoatSNP50 BeadChip v.2.

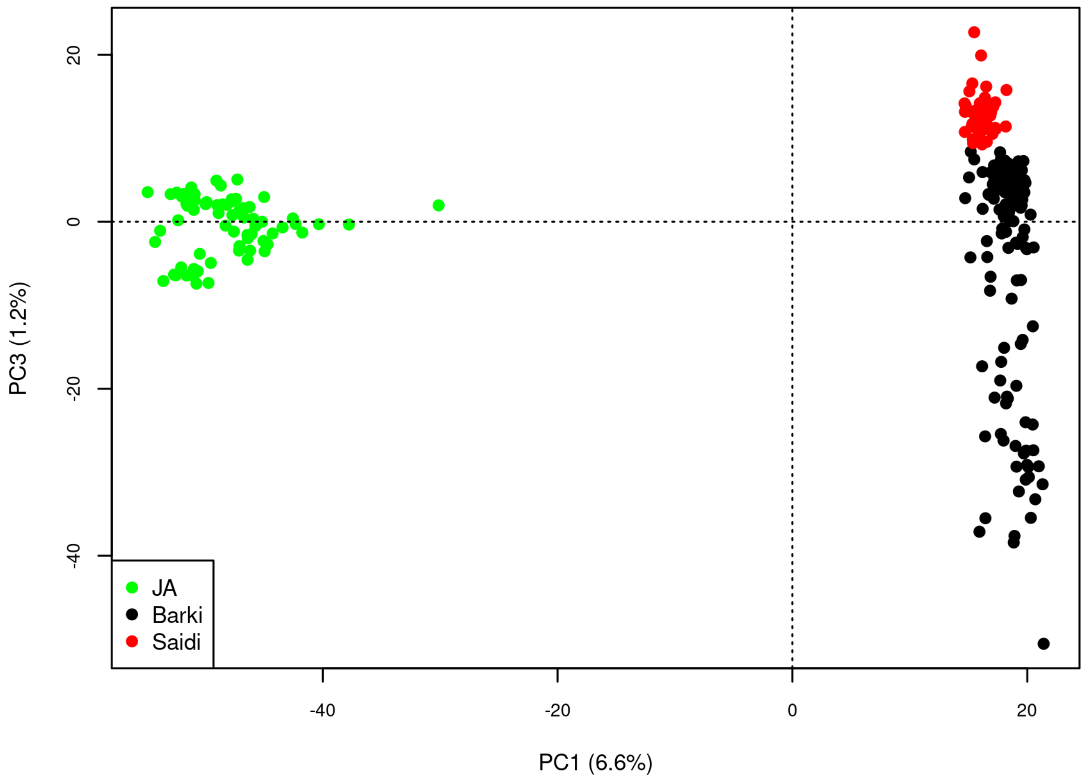

Figure S2: PCA plot of the first and third principal components (PC) on Jabal Akhdar (JA), Barki, and Saidi goat breeds.

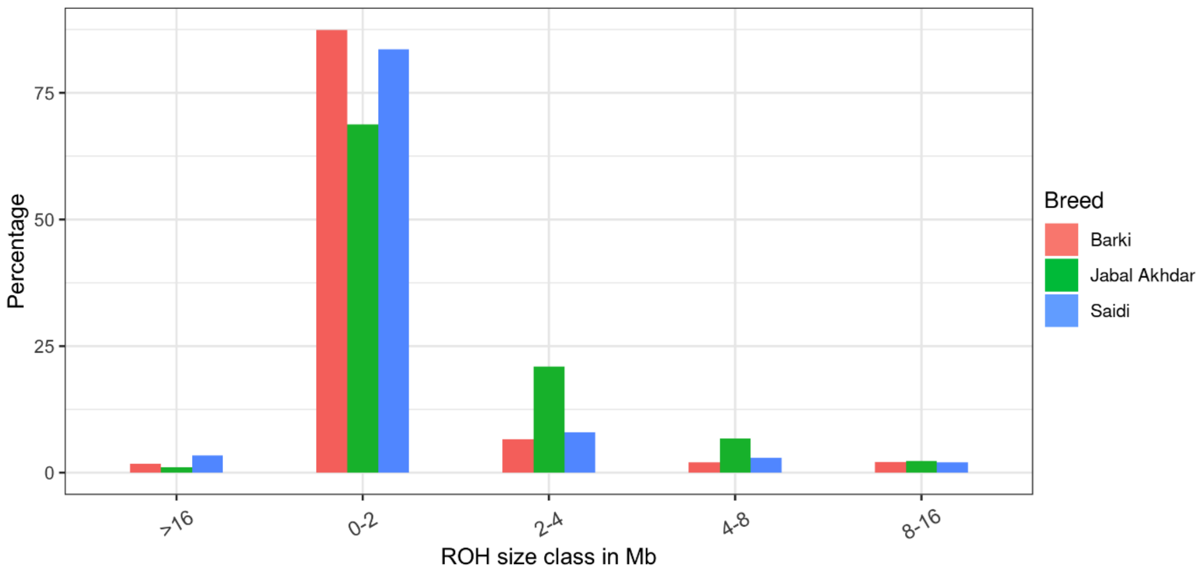

Figure S3: Distribution of the sizes of ROH segments in each breed.

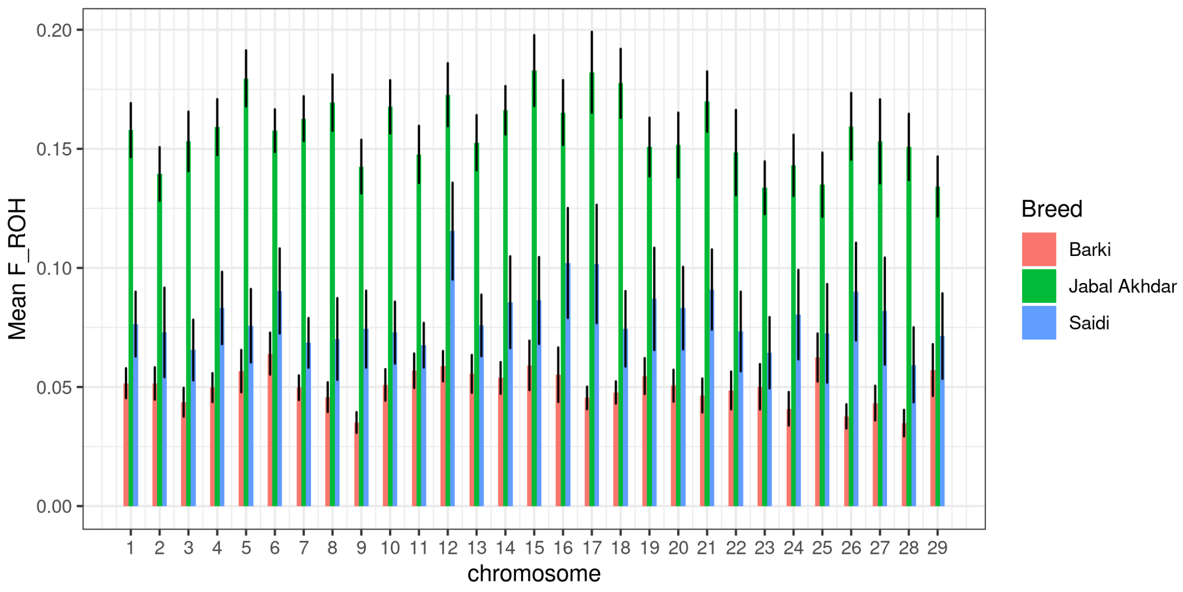

Figure S4: The chromosome-wise inbreeding coefficient (F_{ROH}) for the Jabal Akhdar, Barki and Saidi goat breeds. Data represent mean ± SEM

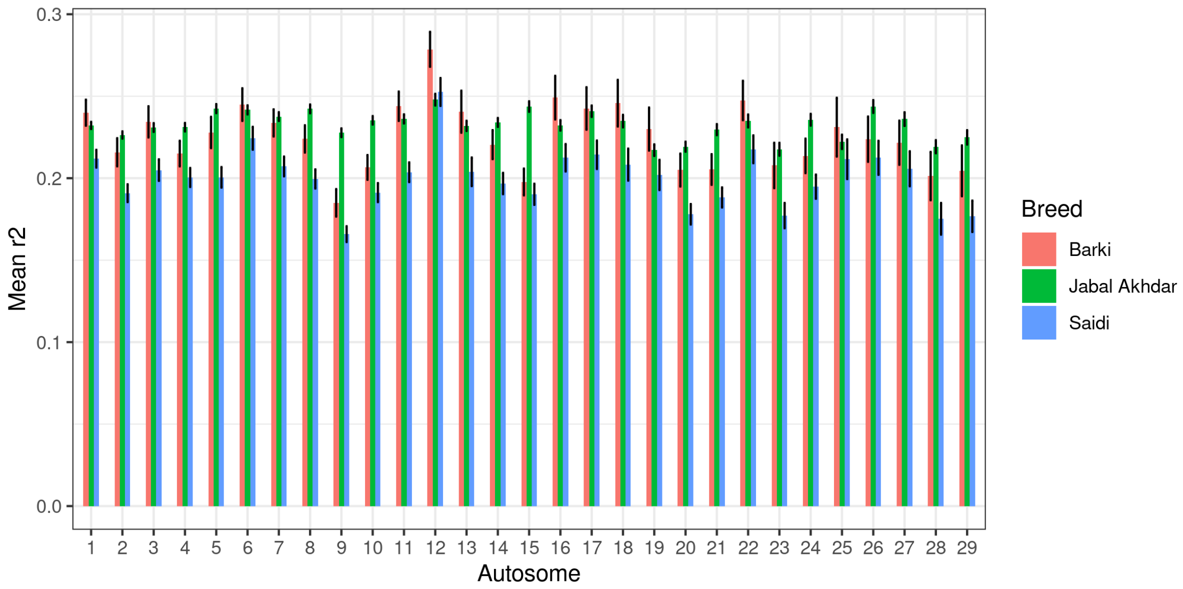

Figure S5: The chromosome-wise square correlation coefficient (r^2) for the Jabal Akhdar, Barki and Saidi goat breeds. Data represent mean ± SEM

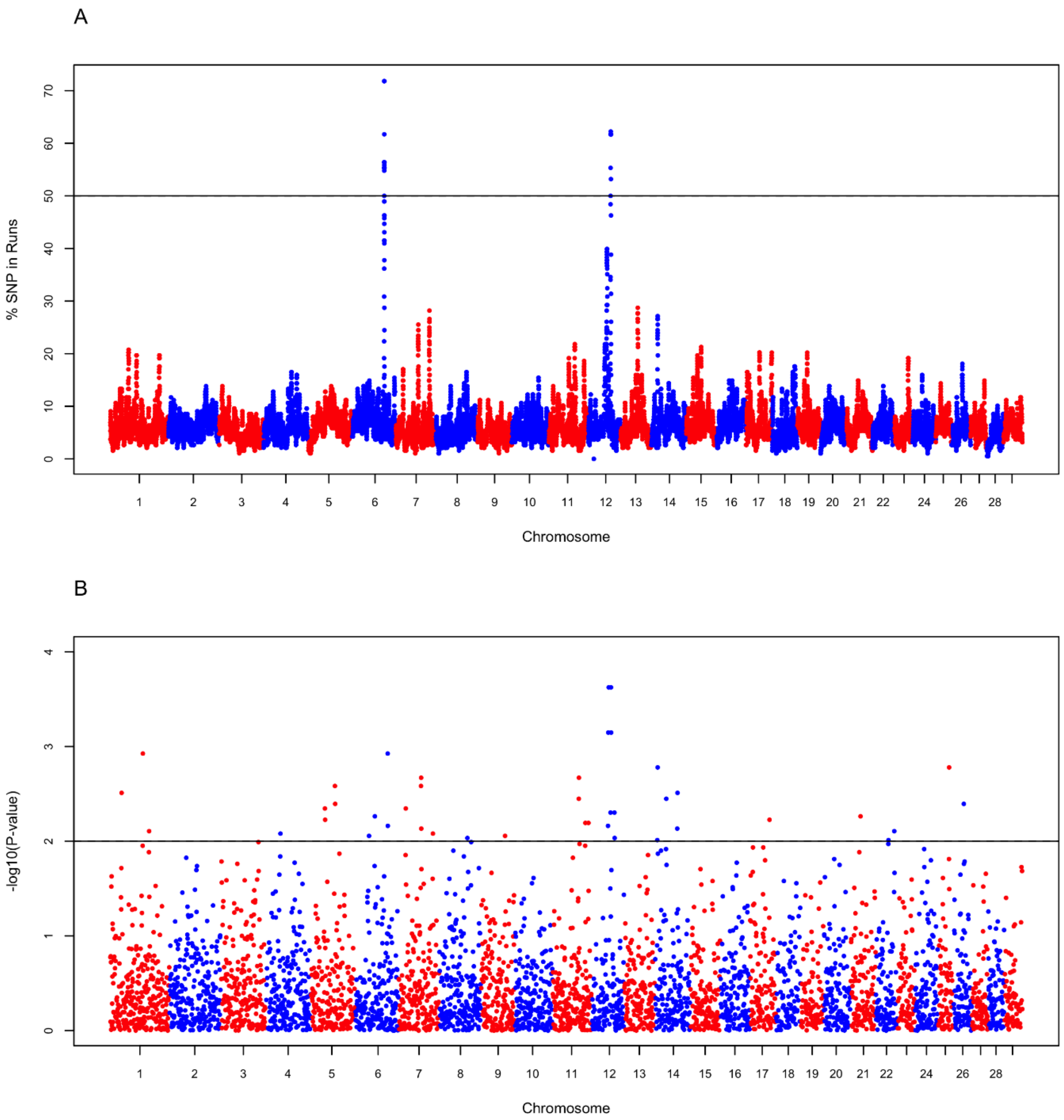

Figure S6: Manhattan plots of the genome-wide (A) ROH islands and (B) *iHS* analysis on combined desert goat breeds (Barki and Saidi). The significance threshold is set on ROH prevalence at 50% of the individuals. For the *iHS* analysis, the significance thresholds are set as $-\log_{10}$ (*P*-value) of 2.

Supplementary Table S1: is published separately - see the supplementary material files.

Supplementary Table S2: is published separately - see the supplementary material files.

Supplementary Table S3: is published separately - see the supplementary material files.

Supplementary Table S4: is published separately - see the supplementary material files.

Supplementary Table S5: is published separately - see the supplementary material files.

Supplementary Table S6: is published separately - see the supplementary material files.

Supplementary Table S7: is published separately - see the supplementary material files.
